# Whey protein consumption after resistance exercise reduces energy intake at a post-exercise meal

**DOI:** 10.1007/s00394-016-1344-4

**Published:** 2016-11-10

**Authors:** Alistair Monteyne, Alex Martin, Liam Jackson, Nick Corrigan, Ellen Stringer, Jack Newey, Penny L. S. Rumbold, Emma J. Stevenson, Lewis J. James

**Affiliations:** 1 School of Sport, Exercise and Health Sciences, Loughborough University, Leicestershire, LE11 3TU UK; 2 Department of Sport, Exercise and Rehabilitation, Faculty of Health and Life Sciences, Northumbria University, Newcastle upon Tyne, NE1 8ST UK; 3 Institute of Cellular Medicine, Human Nutrition Research Centre, Newcastle University, Newcastle upon Tyne, NE2 4HH UK

**Keywords:** Appetite, Energy balance, Weight management, Protein synthesis, Anabolism, Body composition

## Abstract

**Purpose:**

Protein consumption after resistance exercise potentiates muscle protein synthesis, but its effects on subsequent appetite in this context are unknown. This study examined appetite and energy intake following consumption of protein- and carbohydrate-containing drinks after resistance exercise.

**Methods:**

After familiarisation, 15 resistance training males (age 21 ± 1 years, body mass 78.0 ± 11.9 kg, stature 1.78 ± 0.07 m) completed two randomised, double-blind trials, consisting of lower-body resistance exercise, followed by consumption of a whey protein (PRO 23.9 ± 3.6 g protein) or dextrose (CHO 26.5 ± 3.8 g carbohydrate) drink in the 5 min post-exercise. An ad libitum meal was served 60 min later, with subjective appetite measured throughout. Drinks were flavoured and matched for energy content and volume. The PRO drink provided 0.3 g/kg body mass protein.

**Results:**

Ad libitum energy intake (PRO 3742 ± 994 kJ; CHO 4172 ± 1132 kJ; *P* = 0.007) and mean eating rate (PRO 339 ± 102 kJ/min; CHO 405 ± 154 kJ/min; *P* = 0.009) were lower during PRO. The change in eating rate was associated with the change in energy intake (*R* = 0.661, *P* = 0.007). No interaction effects were observed for subjective measures of appetite. The PRO drink was perceived as creamier and thicker, and less pleasant, sweet and refreshing (*P* < 0.05).

**Conclusion:**

These results suggest whey protein consumption after resistance exercise reduces subsequent energy intake, and this might be partially mediated by a reduced eating rate. Whilst this reduced energy intake is unlikely to impair hypertrophy, it may be of value in supporting an energy deficit for weight loss.

## Introduction

Muscle hypertrophy is highly desirable to a wide range of populations, ranging from those seeking optimal athletic performance, to those seeking to maintain functional capacity for health. Concurrently, resistance exercise is also recommended as part of a holistic model for weight management [1]. Resistance exercise and post-exercise protein feeding synergistically potentiate muscle protein synthesis, orchestrating muscle fibre hypertrophy [2]. At least in young, resistance-trained men, whey protein has been shown to stimulate muscle protein synthesis to a greater extent than other proteins when doses of 20–25 g protein are ingested [3], with this amount of whey protein being sufficient to maximise this response after lower limb resistance exercise [4].

Protein has been suggested to be the most satiating macronutrient, and protein feeding at rest has been shown to reduce subsequent energy intake compared to other macronutrients [5], and protein-containing drinks have been shown to attenuate energy intake at a subsequent meal in a dose-dependent manner [6]. Therefore, if post-exercise protein intake reduces subsequent energy intake sufficiently, this might reduce the anabolic response to subsequent protein intake, which is potentiated for some time after exercise [7].

Whilst resistance exercise in isolation has been shown to alter appetite regulation, to date, very few studies have considered the interaction of exercise and post-exercise nutrition on subsequent appetite and energy intake. This is particularly important for resistance exercise where post-exercise protein intake is recommended to maximise the anabolic response [2]. When consumed after aerobic exercise, Clayton et al. [8] observed no significant difference in subsequent energy intake between energy-matched whey protein and carbohydrate drinks. It is feasible, however, that resistance exercise may interact with liquid protein to elicit a dissimilar response to aerobic exercise; a premise that has yet to be investigated.

Therefore, the purpose of this study was to compare drinks containing dextrose (i.e. carbohydrate) and whey protein consumed after resistance exercise on subsequent appetite and energy intake. It was hypothesised that the whey protein drink would suppress appetite and reduce energy intake relative to the carbohydrate drink.

## Methods

### Subjects

After approval by the Loughborough University Ethics Approvals (Human Participants) Sub-Committee, 15 physically active, healthy males, who included resistance exercise in their exercise routine (age 21 ± 1 years, body mass 78 ± 11.9 kg, stature 1.78 ± 0.07 m, BMI 24.6 ± 2.6 kg m^−2^) provided consent and completed this study. Subjects were not restrained, disinhibited or hungry eaters [9]. Subjects performed a familiarisation trial and two experimental trials, with the experimental trials being administered in a randomised double-blind manner and separated by ≥5 days. Using previous data from our laboratory for the main outcome variable (i.e. ad libitum energy intake), an a priori sample size calculation with statistical power of 0.95 and *α* of 0.05 estimated 15 subjects would be required to reject the null hypothesis if there was a mean difference of 400 kJ between trials.

### Familiarisation trial

Subject’s stature and mass were recorded and skinfold measurements were made at four sites (biceps, triceps, subscapular and suprailiac) to estimate body fat using the Siri equation [10]. Subjects then completed a 5-min warm-up on a friction-braked cycle ergometer (Monark828E, Varberg, Sweden), at a standardised work rate (1.5–2 W/kg body mass). One repetition maximum (1RM) was then determined for unilateral leg extension and leg flexion (Technogym Element + Leg Extension and Leg Curl, Technogym U.K. Ltd, Berkshire, UK). A successful repetition was judged by subjects producing an acceptably full range of motion. Subjects rested as required between 1RM attempts. Subjects then completed two sets of 10 reps at 70% of 1RM (Table 1) to familiarise them with the resistance training protocol used in the experimental trials, after which they were familiarised with the ad libitum pasta meal described later.

### Pre-trial standardisation

Subjects completed a food and activity diary in the 24 h preceding the first experimental trial and were asked to replicate this in the 24 h before their second trial. Atypical dietary habits, alcohol ingestion and strenuous physical activity were not permitted in this period. All subjects consumed a standardised breakfast two h before exercise commenced, providing 15% of estimated energy requirements (RMR [11] multiplied by a physical activity level of 1.7) and 1 g/kg body mass of carbohydrate. The breakfast was consumed in the subject’s home and consisted of semi-skimmed milk (Tesco, Cheshunt, UK) and Nutri-Grain bars (Kelloggs, Manchester, UK) in a ratio of 125-ml milk 30 g Nutri-Grain. Compliance with these pre-trial requirements was verbally confirmed prior to each trial.

### Experimental trials

Participants arrived at the testing facility between 10:00 and 11:00 (standardised within subjects), and post-void body mass in minimal clothing was measured. Subjects completed approximately 50 min of resistance exercise and then immediately ingested either a protein (PRO) or carbohydrate (CHO) drink. This was followed by a period of 60-min rest in a comfortable environment. The ad libitum meal was served 65 min after the end of exercise, and subjects were allowed 20 min in which to eat. Questionnaires assessing subjective appetite were collected at regular intervals throughout, along with a drink characteristic questionnaire that was collected after post-exercise drink ingestion.

### Resistance exercise

Subjects completed the standardised 5-min warm-up described for the familiarisation trial, followed by 2-min rest. Resistance exercise was unilateral extension of the right and left leg, followed by unilateral flexion of the right and left leg. For each exercise on each leg subjects completed one warm-up set of 10 repetitions at 35% 1RM and four working sets of 10 reps at 70% 1RM. If subjects fatigued before they had completed four sets of 10 reps during the first experimental trial, they replicated this work in the second trial. In the second trial, all subjects were able to replicate work done from the first trial. Two minutes rest was allowed between each set. Subjects were provided with water ad libitum up until the start of the final exercise (i.e. left leg flexion) during the first trial, with this amount matched during the second trial.

### Ad libitum meal

Subjects were seated in an eating booth to isolate them from external stimuli as much as possible. The test meal consisted of pasta (400 g dry-weight), Bolognese sauce (400 g), and olive oil (32 g) (Tesco, Cheshunt, UK). The meal was homogenous in nature and provided 5.84 ± 0.04 kJ/g (12% protein, 69% carbohydrate, 19% fat). Subjects were initially provided with a portion containing just over half of the total food prepared. A new portion, containing the remainder of the prepared food, was provided part way through the protocol at a time specific to the subjects eating rate. This was to ensure that finishing a bowl did not act as a satiety cue. Subjects were instructed to “eat until comfortably full and satisfied”, at which point they moved from the eating booth to a chair inside the eating laboratory. A period of 20 min was allocated to eat the test meal and subjects remained in the eating laboratory for the entire time. The time spent eating was recorded and together with the total energy intake was used to determine the mean eating rate. Water was available ad libitum during the meal. The meal was served in two large pasta bowls and warmed before serving. All meals were subject to identical preparation, cooking, heating and serving protocols. Food and water intake were measured by weighing bowls and glasses before and after consumption, with energy intake quantified from manufacturer values.

### Post-exercise drink

Subjects were provided with a dextrose monohydrate drink (Myprotein, Manchester, UK) in the CHO trial, and a whey protein isolate drink (WPI90, Volac International Ltd., Orwell, UK) in the PRO trial. (Table 2) The protein drink provided 0.3 g protein/kg body mass, in line with current guidelines [2]. The carbohydrate drink was isoenergetic in comparison with the protein drink, although a little over 0.3 g carbohydrate/kg body mass was provided due the small additional fat and lactose content of the whey protein isolate. Manufacturer values were used to determine the macronutrient and energy content of powders. The powder for each drink was assimilated in 400 ml of no added sugar orange squash (Tesco Stores Ltd., Cheshunt, UK) and the subjects consumed this 400 ml. An additional 100 ml squash was then added to the bottle, mixed with any remaining residue and consumed by the subjects. Subjects were given 5 min to consume the drink. The drink was served in an opaque sports bottle and was consumed through a sports cap to reduce sensory and textural cues. The drink was provided in a randomised, double-blind manner. Drinks were prepared on the same day as the trial, earlier that morning. Subjects were aware that the study was investigating the appetite effects of post-resistance exercise drink composition, but were unaware of the composition of drinks.

At the end of the study, subjects were told that the drinks consumed were a carbohydrate drink and a whey protein drink and were asked if they could identify which drink they had ingested on which trial.

### Subjective appetite questionnaire

Subjects rated their perceptions of appetite via 100-mm visual analogue scales (VAS) [12]. Questions asked were related to hunger “How hungry do you feel?”; fullness “How full do you feel?”; desire to eat (DTE) “How strong is your desire to eat?” and prospective food consumption (PFC) “How much food do you think you could eat?”, with verbal anchors “not at all”/“none at all” at 0 mm and “extremely”/“a lot” at 100 mm. Subjects completed this questionnaire pre-exercise, post-exercise, post-drink, 15 min post-drink, 30 min post-drink, 45 min post-drink, 60 min post-drink and at the end of the test meal. Total area under the curve (AUC) values were calculated for subjective appetite responses in the period between drink consumption and the ad libitum meal (i.e. post-drink to 60 min post-drink).

### Drink characteristics questionnaire

Additional 100-mm VAS questions were assessed immediately after drink consumption. Questions asked were “How pleasant was the drink?”, “How much aftertaste did the drink have?”, “How salty was the drink?”, “How bitter was the drink?”, “How sweet was the drink?”, “How creamy was the drink?”, “How thick was the drink?”, “How sticky was the drink?”, “How fruity was the drink?” and “How refreshing was the drink?”. Verbal anchors “not at all” and “extremely/extreme” were placed at 0 mm and 100 mm, respectively.

### Data analysis

Data were analysed using SPSS 22 (SPSS Inc., Somers, NY, USA). All data were examined for normality of distribution using a Shapiro–Wilk test. Normally distributed data containing one factor were analysed using paired samples *t* tests, and non-normally distributed data containing one factor were analysed using Wilcoxon signed-rank tests. Data containing two factors were analysed using a two-way repeated measures ANOVA. Statistical significance was set at *P* < 0.05. Data are presented as mean ± standard deviation.

## Results

### Pre-trial measurements

There was no difference between trials for pre-trial body mass (PRO 78.9 ± 12.4 kg; CHO 78.7 ± 12.4 kg; *P* = 0.437), or subjective sensations of hunger (PRO 40 ± 20 mm; CHO 40 ± 18 mm; *P* = 0.978), fullness (PRO 51 ± 12 mm; CHO 47 ± 15 mm; *P* = 0.347), DTE (PRO 40 ± 23 mm; CHO 43 ± 21 mm; *P* = 0.193) or PFC (PRO 46 ± 21 mm; CHO 51 ± 19 mm; *P* = 0.282).

**Table 1:** One repetition maximum (1RM) and weight lifted during the working sets (kg) during the resistance exercise in experimental trials

	Right leg extension	Left leg extension	Right leg flexion	Left leg flexion
1RM (kg)	60.7 ± 16.0	60.3 ± 17.0	41.5 ± 9.4	41.5 ± 10.0
Working weight (kg)	42.2 ± 10.4	42 ± 11.3	28.7 ± 5.5	28.7 ± 6.3

Data are mean ± SD

**Table 2:** Composition of post-exercise drinks

	Protein (PRO)	Carbohydrate (CHO)
Volume (ml)	500	500
Energy (kJ)	459 ± 64	459 ± 64
Protein (g)	23.9 ± 3.6	0.4 ± 0.0
Carbohydrate (g)	2.7 ± 0.1	26.5 ± 3.8
Fat (g)	0.1 ± 0.0	0.0 ± 0.0
Fibre (g)	0.4 ± 0.0	0.4 ± 0.0

Data are mean ± SD

**Table 3:** Total area under curve for subjective appetite ratings

Subjective appetite measure	PRO	CHO
Hunger (mm/60 min)	3466 ± 955	3632 ± 813
Fullness (mm/60 min)	2148 ± 921	1982 ± 724
DTE (mm/60 min)	3515 ± 1042	3756 ± 755
PFC (mm/60 min)	3676 ± 906	3922 ± 636

Data are mean ± SD

*DTE* desire to eat, *PFC* prospective food consumption

### Ad libitum meal

**Fig. 1:**
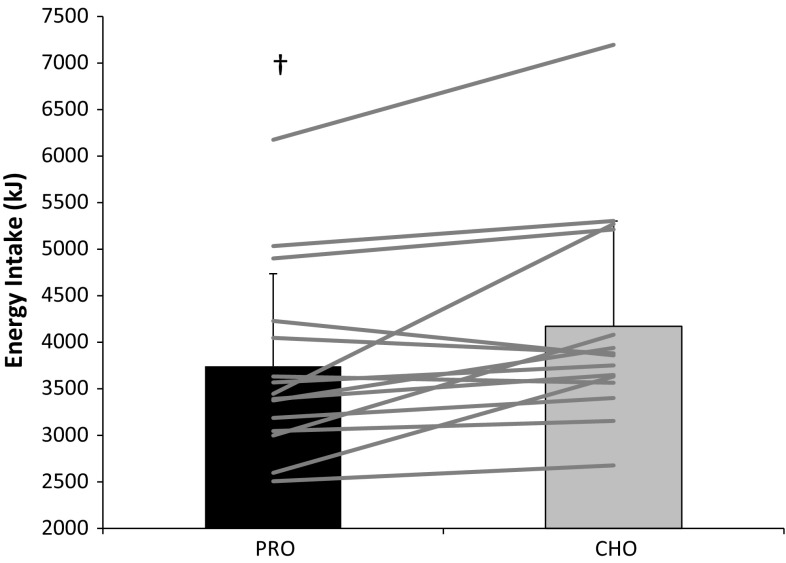
Energy intake at the ad libitum test meal (kJ). *Dagger* (†) significantly different from CHO (*P* = 0.009). *Bars* are mean ± SD, with *lines* representing individual subject data

**Fig. 2:**
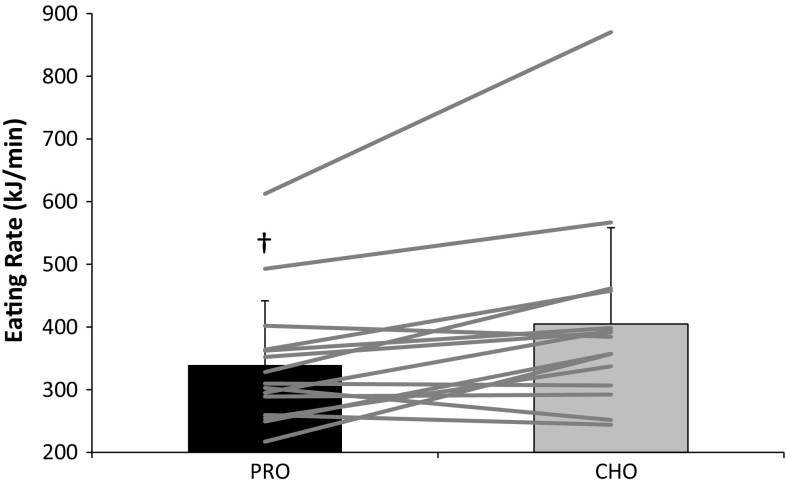
Mean eating rate at the ad libitum test meal (kJ min^−1^). *Dagger* **(†)** significantly different from CHO (*P* = 0.011). *Bars* are mean ± SD, with *lines* representing individual subject data

**Fig. 3:**
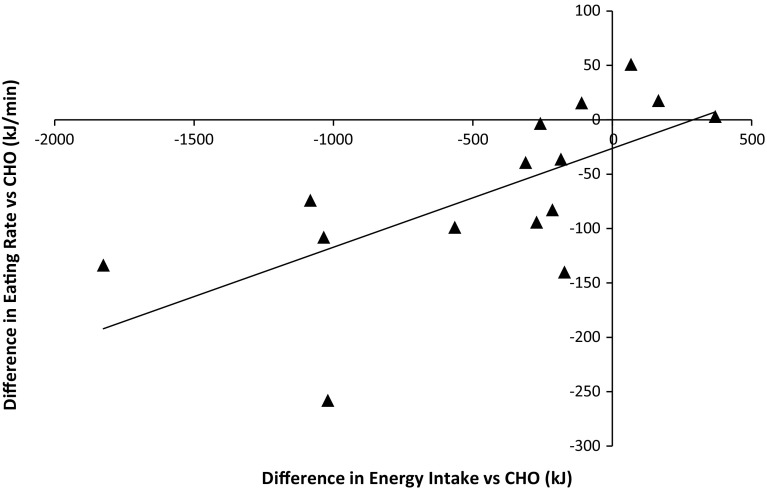
Change in eating rate (kJ min^−1^) versus change in energy intake (kJ) during the ad libitum meal (*r* = 0.662, *P* = 0.007)

Energy intake at the ad libitum meal was reduced during PRO compared to CHO (*P* = 0.009; Fig. 1). Eating rate was also reduced during PRO compared to CHO (*P* = 0.011; Fig. 2). The change in eating rate between trials was associated with the change in energy intake between trials (*r* = 0.662, *P* = 0.007) Fig. 3. Ad libitum water intake did not differ between trials (*P* = 0.691) and amounted to 339 ± 146 ml during PRO and 349 ± 152 ml during CHO. There was no trial order effect for energy intake (trial 1 3874 ± 924 kJ; trial 2 4040 ± 1224 kJ; *P* = 0.599) or eating rate (trial 1 373 ± 90 kJ; trial 2 371 ± 168 kJ; *P* = 0.689).

### Drink perception

**Fig. 4:**
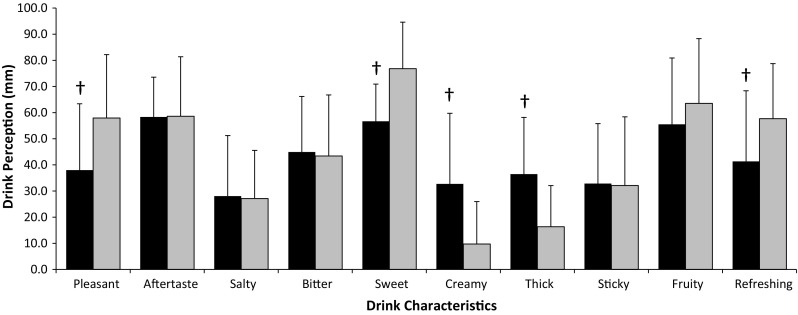
Subjective perceptions of test drinks (mm); PRO (*black square*) and CHO (*grey square*). *Dagger* (†) Significantly different from CHO (*P* < 0.05). *Bars* are mean ± SD

Subjects perceived PRO to be thicker (*P* = 0.001) and creamier (*P* = 0.001) than CHO, whilst CHO was perceived as being more pleasant (*P* = 0.014), sweeter (*P* = 0.004) and more refreshing (*P* = 0.028) than PRO. There was no difference between drinks for any other characteristics (*P* > 0.250, Fig. 4).

### Subjective appetite ratings

There was a main effect of time for all subjective appetite measures (hunger *P* = 0.001; fullness *P* = 0.001; DTE *P* = 0.001; PFC *P* = 0.001), but no main effects of trial (hunger *P* = 0.301; fullness *P* = 0.671; DTE *P* = 0.150; PFC *P* = 0.051) or interaction effect (hunger *P* = 0.559; fullness *P* = 0.442; DTE *P* = 0.163; PFC *P* = 0.302). AUC values in response to the drinks were not different between trials for any subjective appetite variable (hunger *P* = 0.425; fullness *P* = 0.512; DTE *P* = 0.234; PFC *P* = 0.220) (Table 3).

### Detection of study drinks

At the end of the study when subjects were told the drinks used in the study were a carbohydrate drink and a whey protein drink, 11 of the 15 subjects correctly identified on which trial they had consumed which drink.

## Discussion

The aim of this study was to examine the effect of the macronutrient composition of a drink consumed after resistance exercise on subsequent appetite and ad libitum energy intake. The primary finding was that energy intake was reduced after the consumption of a whey protein isolate drink compared to an energy-matched carbohydrate drink. Mean eating rate was also reduced after consumption of the whey protein drink. Furthermore, there were significant differences in drink characteristics, with the whey protein drink being perceived as thicker and creamier, as well as less sweet, pleasant and refreshing, which might have influenced subsequent energy intake.

It has been suggested that the daily discrepancy between intake and expenditure causing long-term weight gain is slight [13, 14]. Accordingly, the modest reduction in energy intake observed in the current study (430 ± 579 kJ) may augment the effects of resistance exercise in aiding long-term weight management. The coefficient of variation of a single-item ad libitum meal with prior dietary standardisation has been shown to be ~8.9% [15]. The mean difference in energy intake between trials in the present study equated to 10.3%, which was slightly greater than that reported by Gregersen et al. [15], although the reproducibility of the ad libitum meal in the present study might have been improved by the inclusion of a familiarisation trial to habituate subjects to the meal and eating environment. Resistance exercise increases acute energy expenditure [16] and the resultant increase in muscle mass [17] might increase daily energy requirements via alterations in basal metabolic rate. The present study suggests that a reduction in energy intake following resistance exercise with whey protein consumption may offer an additional mechanism through which body re-composition might occur.

Several plausible explanations exist as to why protein in drink form might be more satiating than carbohydrate. These include effects on: gastrointestinal appetite-related hormones; circulating amino acids; and the sensory profile of the drink. Protein consumption has been shown to elevate peripheral concentrations of the anorexogenic hormones CCK and GLP-1 to a greater extent than carbohydrate, resulting in greater satiety [18, 19], although the strength of this relationship remains unclear. Additionally, the hyperaminoacidemia that occurs following protein ingestion may affect appetite both directly through amino acid-mediated mechanisms and indirectly by influencing glucose homeostasis [20]. These blood-based measurements were not made in the present study, representing a limitation that should be rectified in future studies. Whilst energy intake was reduced during the PRO trial, there was no difference between trials for any subjective appetite measures. Some [6, 19, 21, 22], but not all [18, 23] previous studies at rest have reported enhanced satiety after consuming protein-containing drinks, but perhaps the inclusion of resistance exercise in the present study, which alters subjective appetite responses independently [16] accounts for the lack of difference observed. In line with this hypothesis, no difference in subjective appetite has been observed following manipulation of the carbohydrate and protein content of drinks consumed after endurance exercise [8, 24], which also independently alters subjective appetite [16].

The greater thickness and creaminess of the protein drink may have played a role in reducing energy intake. The sensory characteristics of a drink modify its satiating properties and might influence subsequent energy intake [25]. Viscosity, or thickness, seems to play a particularly important role, with thicker drinks enhancing expectations of satiety [26, 27]. Within the literature that has noted differences in energy intake between drinks of differing macronutrient content, it is not uncommon for drinks to either differ in hedonic qualities or for subjects to clearly identify differences between drinks in terms of texture or flavour [6, 21, 23, 24]. Bertenshaw et al. [28] demonstrated that matching high protein and carbohydrate drinks for perceived thickness and creaminess resulted in very similar satiety responses, despite liquid protein typically being found to induce greater satiety elsewhere in the literature. Furthermore, protein drinks that were less thick and creamy, despite being matched for nutritional content, were found to be less satiating, resulting in greater ad libitum energy intake compared to a sensory-enhanced protein drink. These results suggest that the sensory characteristics of drinks are critical in determining short-term satiety [28]. The exact mechanisms by which orosensory characteristics of drinks influence appetite and energy intake are not clear, although such factors have been shown to elicit a hormonal effect associated with appetite control [29].

Within the current study, the protein drink was perceived to be thicker and creamier than the carbohydrate drink, and less pleasant. Consequently, it is probable that orosensory factors may have played a causal role in the reduction in energy intake after consumption of the protein drink compared to the carbohydrate drink. Clayton et al. [8] reported energy intake 60 min after consuming whey protein and carbohydrate drinks was not different, whilst Rumbold et al. [24] reported reduced energy intake 60 min after consuming skimmed milk compared to a carbohydrate drink. Interestingly, Clayton et al. [8] did not observe differences in the thickness or creaminess of the drinks, a finding that is likely due to the nature of the whey protein used and the fact that drinks were consumed through a straw to limit orosensory exposure. Whilst Rumbold et al. [24] did not report the subjects sensory perceptions of the drinks, the nature of the drinks (milk vs. orange juice) means it is highly likely that sensory differences, particularly thickness and creaminess would have been present [23]. Collectively, these results suggest that similar to consumption at rest [28], the orosensory effects of drinks consumed after exercise might be important for how a drink impacts ad libitum energy intake. Whilst the failure to match drinks for orosensory factors might represent a limitation of the present study, it also increases the external validity of the study as in practice protein and carbohydrate drinks consumed in a post-exercise setting would likely differ hedonically.

The results of the present study suggest that the reduction in energy intake after protein consumption appears to be at least partially mediated by a reduction in eating rate. Mean eating rate was reduced after protein consumption, and the change in eating rate was associated with the change in energy intake. Empirical evidence suggests that manipulating eating rate affects energy intake, with slower eating rates reducing energy intake [30]. Furthermore, reductions in energy intake as a result of slowed eating rates are not associated with increased hunger, decreasing the risk of subsequent compensatory eating [31]. In evaluation, consuming a protein drink after resistance exercise may be an effective behavioural strategy to modify subsequent eating rate, which in turn might reduce energy intake without deleterious effects on hunger.

Resistance exercise increases muscle protein synthesis [32], and protein feeding post-exercise further potentiates this response [33], whilst concurrently suppressing muscle protein breakdown [34]. The synergistically stimulated increase in muscle protein synthesis, and to a lesser extent decrease in muscle protein breakdown, permits positive net protein balance and consequent muscle fibre hypertrophy [17]. Whilst the impact on hypertrophy of the reduction in energy intake observed in the present study is unknown, it seems unlikely it would significantly impair the process. Longland et al. [35] restricted energy intake by ~40% during a 4-week resistance training period whilst providing protein equivalent to 1.2 and 2.4 g/kg body mass in two separate groups, respectively. Over the 4-week training period both groups lost ~3.5 kg of body mass, but there was no change in lean mass in a group consuming 1.2 g/kg protein and a ~1.2 kg increase in lean mass in a group consuming 2.4 g/kg protein. This suggests lean mass can be augmented whilst in negative energy balance, providing a high protein intake and resistance exercise are in place, at least in non-resistance-trained males. The reduction in energy intake after the whey protein drink in the present study equates to ~3% of subject’s estimated daily energy requirements. Given the findings of Longland et al. [35], the small reduction in energy intake observed after the whey protein beverage in the present study is unlikely to adversely affect the augmentation of lean mass.

The proximity of the ad libitum meal to the post-exercise drink is relatively close within the current investigation, and it would be interesting to see whether the reduction in energy intake would remain at a more distal time point. The average time interval for voluntary meal requests has been suggested to occur ~80 min after the termination of exercise [36], which is similar to the 65 min used in the present study. Furthermore, the present study only examined a single post-exercise meal and as such future investigations should track energy intake responses over longer periods, as well as including measurements of other components of energy balance (i.e. resting and physical activity energy expenditure). Finally, as the subjects used in the present study were experienced with resistance exercise, these results might not translate to those at the start of a resistance training programme.

To conclude, when a whey protein isolate drink was consumed after resistance exercise in lean men experienced with resistance exercise, in an amount known to maximise muscle protein synthesis, there was a reduction in subsequent energy intake at a single ad libitum meal compared to an energy-matched carbohydrate drink. The reduction in energy intake was modest (430 kJ), and may have been partially mediated by a reduction in eating rate, as well as the sensory characteristics of the drink. Whilst this reduction in energy intake is unlikely to impair the energy provision required to optimise muscle hypertrophy, it may be beneficial for those individuals seeking to reduce body fat.
